# In *BCR-ABL1* Positive B-Cell Acute Lymphoblastic Leukemia, Steroid Therapy Induces Hypofibrinogenemia

**DOI:** 10.3390/jcm11071776

**Published:** 2022-03-23

**Authors:** Elisa Buzzatti, Fabio Forghieri, Giovangiacinto Paterno, Francesco Marchesi, Chiara Sarlo, Fabio Giglio, Nicola Fracchiolla, Mariarita Sciumè, Raffaele Palmieri, Fabiana Esposito, Luca Guarnera, Lisa Mercante, Maria Rosaria Pascale, Flavia Mallegni, Arianna Savi, Vittorio Forte, Luca Maurillo, Francesco Buccisano, Adriano Venditti, Maria Ilaria Del Principe

**Affiliations:** 1 Hematology, Department of Biomedicine and Prevention, University Tor Vergata, 00133 Rome, Italy; buzzattielisa@gmail.com (E.B.); g.paterno@aol.com (G.P.); raffaele.f.palmieri@gmail.com (R.P.); fabiana.e91@gmail.com (F.E.); lucaguarnera@live.com (L.G.); lisa.mercante@gmail.com (L.M.); maryroses91@hotmail.it (M.R.P.); flaviamallegni@gmail.com (F.M.); arianna.savi@gmail.com (A.S.); francesco.buccisano@uniroma2.it (F.B.); dlpmlr00@uniroma2.it (M.I.D.P.); 2 U.O.S.D. Mieloproliferative, Fondazione Policlinico Tor Vergata, 00133 Rome, Italy; vittorio.forte@ptvonline.it (V.F.); luca.maurillo@ptvonline.it (L.M.); 3 Section of Hematology, Department of Medical and Surgical Sciences, University of Modena and Reggio Emilia, AOU Policlinico, 41124 Modena, Italy; fabio.forghieri@unimore.it; 4 Hematology and Stem Cell Transplant Unit, IRCCS Regina Elena National Cancer Institute, 00144 Rome, Italy; francesco.marchesi@ifo.it; 5 Hematology, University Campus Biomedico, 00128 Rome, Italy; c.sarlo@unicampus.it; 6 Hematology and Bone Marrow Transplantation Unit, IRCCS San Raffaele Scientific Institute, 20132 Milan, Italy; giglio.fabio@hsr.it; 7 UOC Ematologia, Fondazione IRCCS Ca’ Granda Ospedale Maggiore Policlinico, 20122 Milan, Italy; n.fracchiolla@policlinico.mi.it (N.F.); mariarita.sciume@policlinico.mi.it (M.S.)

**Keywords:** acute lymphoblastic leukemia, hypofibrinogenemia, steroids, *BCR-ABL1* positivity

## Abstract

Hypofibrinogenemia (HF) in adult acute lymphoblastic leukemia (ALL) of B lineage is uncommon and mostly associated with asparaginase (ASP) delivery. Since we noticed a significant reduction in fibrinogen (FBG) plasma levels even before the first ASP dose, we aim to assess the levels of FBG during induction treatment and explore if the FBG fall correlated with therapies other than asparaginase and/or specific leukemia biological features. We retrospectively analyzed FBG levels in 115 patients with B-ALL. In 74 (64%) out of 115 patients FBG decline occurred during the steroid prephase. In univariate analysis, such a steroid-related HF was significantly associated with *BCR-ABL1* rearrangement (*p* = 0.00158). None of those experiencing HF had significant modifications of liver function tests during induction treatment. Our retrospective study suggests that in B-ALL, steroid therapy can also induce HF and that such an event is preferentially observed in patients carrying *BCR-ABL1* rearrangements. The pathogenesis of this phenomenon is still unclear. We attempt to explain it by applying the International Society of Thrombosis and Hemostasis-Disseminated Intravascular Coagulation score (ISTH-DIC score); nonetheless additional studies are needed to clarify further the mechanisms of HF in this subset of patients.

## 1. Introduction

Isolated hypofibrinogenemia (HF) is not a common finding in acute leukemia, being most frequently associated with other abnormalities in coagulation tests due to consumption as in disseminated intravascular coagulation (DIC) caused by cancer or sepsis [1]. Moreover, low fibrinogen (FBG) levels are observed in patients with congenital FBG abnormalities such as type I afibrinogenemia, a rare hereditary coagulation defect in which the genes responsible for production are unable to make a functional glycoprotein because of an inherited mutation. Since FBG synthesis occurs in the liver, hepatic injury is also a possible cause of acquired HF [2]. Among subtypes of acute leukemias, acute promyelocytic leukemia (APL) is known to be associated with HF, which results from DIC or primary fibrinolysis [3]. In acute lymphoblastic leukemia (ALL) isolated HF has most commonly been attributed to asparaginase (ASP) delivery. Lymphoid leukemic-cell survival is greatly dependent on an optimal asparagine supply. ASP is a chemotherapeutic agent, widely used in ALL induction regimen, which depletes asparagine reserves, therefore leading to cell death. ASP is known to cause hemostasis impairment by reducing the synthesis of clotting factors, especially FBG, which may cause severe bleeding. On the other hand, ASP inhibits the synthesis of antithrombin III (AT III) leading to an increased risk of thrombosis [4,5]. Therefore, HF is a laboratory abnormality that, not uncommonly, can be found during induction-remission treatment of ALL [6,7]. In our patients diagnosed with ALL, we have noticed significant reductions in FBG plasma levels not only after ASP administration but also before delivering it, or even in some patients who did not receive it.

Based on these findings, the aims of our retrospective study are to: (1) identify patients with newly diagnosed B-lineage ALL (B-ALL), who experienced a decrease in plasma FBG levels; (2) establish in which phase of the treatment HF developed; (3) analyze the possible correlation of HF with demographic and biologic features of B-ALL. We also analyzed coagulation tests during the treatment period and calculated the International Society of Thrombosis and Hemostasis-DIC score (ISTH-DIC score) [8], in an attempt to hypothesize mechanisms underlying HF. Finally, we evaluated the clinical impact of HF in these patients and the effectiveness of the prophylactic strategies that we adopted.

## 2. Design and Methods

### 2.1. Selection of Patients

We retrospectively evaluated clinical and laboratory data of 115 consecutive patients diagnosed with B-ALL between 2008 and 2020, in six Italian Hematology Centers located in Rome, Milan and Modena. Information was documented using a case report form and all sensitive data were anonymized. Analyzed variables included the following baseline parameters: gender, age, genetic/cytogenetic features, type of *BCR-ABL1* transcript, white blood cell count (WBCc), bone-marrow (BM) blast-cell infiltration and presence of extramedullary disease. Information about date of diagnosis, date of initiation of steroid prephase, chemotherapy (CHT) or tyrosine kinase inhibitors (TKI), day of FBG decrease and duration of this event were also collected. Moreover, we assessed whether any episodes of thrombosis or bleeding occurred during this period and whether any treatment was instituted. HF was treated either with fresh frozen plasma (FFP) or with FBG concentrate. Patients with sepsis at diagnosis or with a positive personal history for liver diseases or with a positive personal/family history of congenital HF were excluded from the study.

### 2.2. Laboratory Parameters

Coagulation tests were assessed at diagnosis, at the beginning of the steroid prephase, at the start of CHT/TKI and then at least twice a week before ASP administration; the date of the lower value of FBG was noted in the CRF. We assessed plasma FBG levels with Clauss method, prothrombin time (PT) and International Normalized Ratio (INR), D-dimer and AT III levels. We calculated DIC score according to ISTH as follows: platelet count: >100 × 10^9^/L = 0, <100 × 10^9^/L = 1, <50 × 10^9^/L = 2; D-dimer: <500 ng/mL (no increase) = 0, between 500 and 4000 ng/mL (moderate increase) = 2, >4000 ng/mL (strong increase) = 3; FBG: >100 mg/dl = 0, <100 mg/dl = 1, and prolonged PT: <3 s = 0, >3 s but <6 s = 1, >6 s = 2. The normal range of FBG was 200–400 mg/dl and for D-dimer <500 ng/mL. Patients with ISTH DIC score ≥ 5 were considered to have overt DIC. Liver function tests (LFTs) [Albumin level (g/dL), serum glutamic oxaloacetic transaminase (GOT) (U/L), serum glutamin pyruvic transaminase (GPT) (U/L), alkaline phosphatase (ALP) (UI/L)] were performed after the steroid prephase on the day of the start of CHT/TKI. Any laboratory deviation from normal was graded according to the *Common Toxicity Criteria for Adverse Events version 5 (27 November 2017).* In this scale, FBG decrease was graded as follows: grade (G)1 = <1.0–0.75 × LLN (lower limit of normal) or, if abnormal, <25% decrease from baseline; G2 = <0.75–0.5 × LLN or, if abnormal, 25–<50% decrease from baseline; G3 = <0.5–0.25 × LLN or, if abnormal, 50–<75% decrease from baseline; G4 = <0.25 × LLN or, if abnormal, 75% decrease from baseline or absolute value <50 mg/dL.

### 2.3. Statistical Analysis

Categorical data were descripted as numbers with percentages, while continuous variables with medians (range). We considered the variable “age” as a continuous variable, but we also categorized it in two groups basing on the cutoff value of 65 years. The comparison of dichotomous variables was performed using the χ^2^ test with Yates’ correction and Fisher’s exact test; for continuous variables the nonparametric Mann–Whitney U test was used. To compare the data of more than two groups the Kruskal–Wallis test was used. A *p*-value less than <0.05 was considered statistically significant (2-tailed). The data were analyzed using NCSS 10 Statistical Software. NCSS, LLC. Kaysville, UT, USA.

## 3. Results

### 3.1. Patients Characteristics

The clinical and laboratory characteristics of the 115 patients at diagnosis are summarized in Table 1. A total of 55 (48%) patients were females and 60 (52%) were males with a median age at diagnosis of 56 years (range 18 to 89 years). No patient had a positive history of any congenital FBG disorders or acquired liver disease. Median BM blast-cell infiltration at diagnosis was 90% (range 20 to 100%) with a median WBCc of 11.8 × 10^9^/L (range 0.840–407 × 10^9^/L). Thirty-eight (33%) patients had an extramedullary disease. The patients were categorized, according to the presence or absence of *BCR-ABL1* transcript, as *BCR-ABL1*-negative (*n* = 52–60%) and *BCR-ABL1*-positive (*n* = 48–55%). In 51 (92%) of 55 *BCR-ABL1*-positive patients, we collected information about the fusion protein isoform, which was p190 transcript in 38 (70%) and p210 in 10 (18%). Three (5%) patients had a concomitant expression of p210 and p190 transcript. *BCR-ABL1*-positive patients were treated with TKI with (*n* = 12–22%) or without CHT (*n* = 43–78%). Fifty-four (90%) of *BCR-ABL1*-negative patients were treated with intensive CHT according to the current Gruppo Italiano Malattie EMatologiche dell’Adulto (GIMEMA) protocols (LAL0904, LAL1104, LAL1308, LAL1913) [9,10], while the remaining six (10%) were treated according to the Northern Italy Leukemia Group (NILG) protocol (NILG-ALL 10/07) [11], R-Hyper-CVAD regimen [12], or CODOX-M/IVAC regimen [13]. In 12 (20%) cases the protocols did not include ASP during the treatment.

### 3.2. Fibrinogen Fluctuations

**Figure 1:**
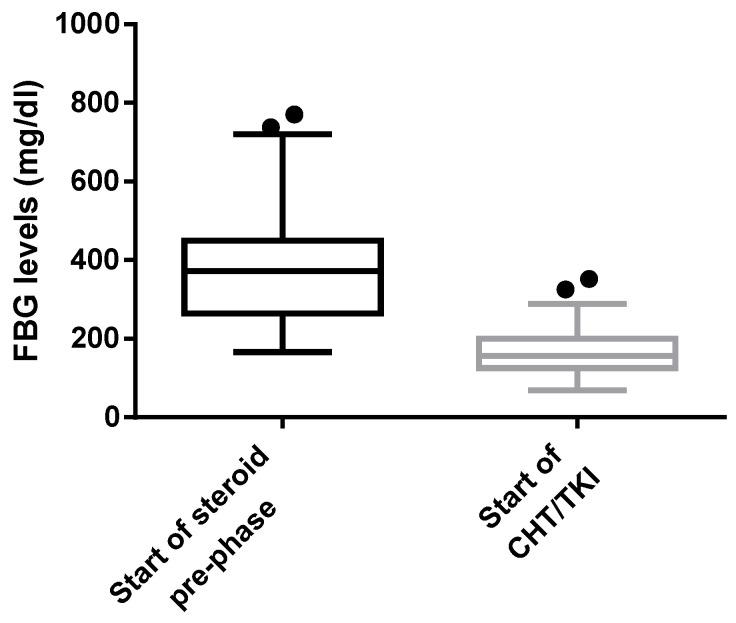
Whiskers plot representation of overall drop of FBG levels in the 74 patients who experienced HF; the two boxes refer to two timepoints: at the start of steroid prephase and at the start of CHT/TKI. The dots outside the whiskers represent the outliers. The difference between the two groups using Mann–Whitney U test is statistically significant (*p* < 0.0001). FBG: fibrinogen; HF: hypofibrinogenemia; CHT: chemotherapy; TKI: tyrosine kinase inhibitors.

At diagnosis, before the start of any treatment, we observed a G1 HF in 6 patients (5%—2 *BCR-ABL1*-positive and 4 *BCR-ABL1*-negative). After a median of 7 days (range 3 to 28) from steroid initiation, a fall in FBG plasma levels was observed in 74 patients (64%) showing a statistically significant difference between FBG levels at the start of the steroid prephase and at the start of CHT/TKI (*p* < 0.0001) [Figure 1].

FBG declined by a median of 54% (range 24 to 86%). Overall, during induction treatment, a total of 87 patients (75%) showed HF. Due to the different disease biology and therapeutic approaches, we decided to analyze data from *BCR-ABL1*-positive and negative patients separately. Fifty-three of 87 (60%) patients developing HF were *BCR-ABL1* positive, accounting for 96% of all *BCR-ABL1* positive cases (53/55). Forty-four (83%) of 53 patients developed HF after a median of 7 days (range 4 to 28) from steroid prephase initiation. In 21 (48%), HF was G1–2 and G3–4 in the remaining 23 (52%). Nine patients (16%) developed a G3–4 HF while receiving TKI therapy. For the *BCR-ABL1*-negative subset, a total of 34/60 (57%) patients showed a FBG decrease before the administration of ASP. Again, most patients experienced HF during the steroid prephase as it was detected in 30/60 (50%) patients, after a median of 7 days (range 3 to 15) from steroid initiation. Among them, 13 (43%) patients had a G1–2 HF and 17 (57%) a G3–4. Four patients (7%) developed HF after the start of CHT but before ASP delivery (1 patient G2; 3 patients G3) [Table 2].

In 12 (20%) *BCR-ABL1*-negative patients, HF occurred after ASP delivery. Furthermore, no thrombotic events occurred in either group.

### 3.3. Univariate Analysis

We explored the correlation between HF and features of B-ALL. Overall, during the steroid prephase, there was no significant correlation with gender (*p* = 0.12), WBCc (*p* = 0.36), extramedullary disease (*p* = 0.44), BM blast count (*p* = 0.31) or days of steroid prephase (*p* = 0.25). For age, we found no correlation either when we considered the variable as continuous (*p* = 0.27) or when we used the cutoff value of 65 years (*p* = 0.26) [Table 3].

Overall, no significant alteration of liver function tests was detected. We collected LFTs at one timepoint: at the start of CHT/TKI post-steroid prephase. For GOT, GPT, alkaline phosphatase and albumin, there was no statistically significant difference between patients who showed HF and those who did not (*p* = 0.87, *p* = 0.66, *p* = 0.97, *p* = 0.74). [Figure S1A–D].

We also compared INR at the three timepoints using the Kruskal–Wallis test: no statistically significant difference was found between the levels of this test before and after the use of steroids (*p* = 0.89) [Figure S2.]

Nevertheless, FBG decrease had a significant correlation with *BCR-ABL1* positivity both in the steroid prephase (*p* = 0.00158) [Figure S3] and during the entire induction course (*p* < 0.0001) [Figure S4]. No correlation was found between HF during the steroid prephase with the *BCR-ABL1* isoform (p190 *p* = 0.71; p210 *p* = 0.63; p190/p210 *p* = 0.89) or with the remission rate (*p* = 0.99).

### 3.4. ISTH-DIC Score

Based on the coagulation test performed during induction treatment, we calculated the ISTH-DIC score at three different time points: at diagnosis, at the start of the steroid prephase and on the first day of CHT/TKI treatment. For the *BCR-ABL1*-positive group, we were able to collect these data in 46/53 (87%) patients who experienced HF. Of these, 35 (76%) did not show a substantial modification of their DIC score, 4 (8.7%) had a DIC score ≥ 5 at diagnosis, 7 (15.3%) patients had a DIC score ≥ 5 at the time of chemotherapy/TKI initiation. The difference between the DIC score at the start of the steroid prephase and DIC score on the first day of CHT/TKI, was not statistically significant (*p* = 0.26) [Figure S5].

For the *BCR-ABL1*-negative group, we collected data in 29 of the 34 patients who experienced HF before the ASP dose. A total of 28 (97%) of them did not show a significant modification of DIC score, while 1 (3%) developed an overt DIC at the third time point of evaluation (*p* = 0.67) [Table 4].

### 3.5. Prophylaxis of Hemorrhagic Events

Recommendations for bleeding prophylaxis were based on the most recent GIMEMA protocol LAL1913, which considers FBG level < 100 mg/dl as a critical threshold, requiring appropriate intervention. However, the decision whether or not to start prophylaxis was a medical judgement also based on clinical conditions. In the *BCR-ABL1*-positive group the physician decided to correct HF in 25 patients: in 18 using FFP while in 7 patients using FBG concentrate. In 3 patients, in whom it was decided not to correct HF despite the level of FBG, no significant bleeding occurred. In one case, FFP was used with a FBG level > 100 mg/dL by medical decision, despite no evidence of bleeding. Overall, only one patient (0.018%) experienced an episode of bleeding during this period but with a level of FBG > 100 mg/dL, and by medical decision he did not receive FFP. Among the *BCR-ABL1*-negative group, in 13/60 (21%) patients, the level of FBG reached a critical value before ASP delivery. A total of 5 patients received a prophylactic infusion of FFP; 6 patients received FBG concentrate; and in 2 patients, despite the level of FBG, it was decided not to treat HF and no bleeding was detected. Overall, only one patient (0.017%) experienced an episode of hemorrhage of an unknown grade with a level of FBG < 100 mg/dl despite an FFP infusion, but this complication occurred after the administration of ASP.

## 4. Discussion

The first purpose of the present analysis was to identify and describe the characteristics of the patients with B-ALL who experienced HF during induction treatment. At diagnosis, 6 patients (5%) already showed a low FBG level of grade 1, which was not attributable to any congenital or acquired fibrinogen disorders [14]. A similar incidence of HF at diagnosis was previously reported in a series of 187 ALL patients, and the cause of this event has not been explained [15]. On the contrary, during induction treatment, we observed HF as a frequent finding, occurring in 75% of the patients. We found no correlation between HF and gender or age. Sciumè et al. analyzed a small cohort of 21 patients with 17 patients ≤ 65 years and 4 patients > 65 years and reported that HF was found in 3 out of 4 elderly patients [16]. The tendency of FBG to decrease in patients of >65 years was therefore emphasized, but in our larger case series this finding was not confirmed. Instead, we found that the status of *BCR-ABL1* positivity can be associated with the development of HF. Indeed, in this setting we observed an increased tendency to develop HF, which occurred early, during the steroid prephase, and persisted during the induction treatment. No correlation was found between HF and the *BCR-ABL1* fusion protein isoform, perhaps because in ALL the p190 subtype prevails over p210 subtype or p190/210, and our findings line up with the literature [17]. A recent study identified the p210 isoform as a strong predictor of HF among patients with *BCR-ABL1*-positive B-ALL [18], but the data analyzed referred to FBG state at diagnosis. In our cases, the two *BCR-ABL1*-positive patients who manifested HF at diagnosis showed concomitant expressions of p190 and p210 isoform.

To demonstrate the lack of influence of LFTs on HF, we collected these data at the start of CHT/TKI post-steroid prephase and compared them between patients experiencing HF and those who did not, showing no statistical correlation [Figure S1A–D]. Additionally, to reinforce the hypothesis that HF in our study could not be associated to liver failure because of the reduced production of vitamin k-dependent coagulation factors, we showed that INR values did not change significantly at the three timepoints in patients with HF [Figure S2].

To explore the underlying mechanism of HF, we calculated the ISTH-DIC score, the most widespread score to assess impending or overt DIC. However, this is not standardized for acute leukemia, since at least one parameter of the index has a poor value in this setting of patients [19]. In fact, thrombocytopenia can be a consequence of an altered coagulation, but in acute leukemia, it also reflects the degree of bone-marrow blast infiltration. In addition, its reliability can be altered by the frequent platelet-transfusion support required in these patients.

We observed that among *BCR-ABL1*-positive patients, there was not a statistically significant difference between the DIC score at the start of the steroid prephase and the DIC score on the first day of CHT/TKI. Two studies by Sarris et al. showed that 67–78% of adult ALL patients developed DIC during induction therapy [6,20]. However, in our series, several patients had HF without any modification of D-dimer or other coagulation tests, and in those with higher level of D-dimer, there was no substantial modification of ISTH-DIC score during the treatment. Therefore, we assumed that HF was a direct effect of primary fibrinolysis rather than of thrombin activation as in DIC. In DIC, the physiological balance between clotting and clot lysis is disrupted, with the activation of both thrombin and plasmin, while primary fibrinolysis is the result of plasmin cleavage of FBG or soluble fibrin [21].

D-dimer is a specific marker of DIC, since its rise means that thrombin has proteolyzed FBG to form fibrin, which has been cross-linked by factor XIII activated by thrombin and then proteolyzed by plasmin to liberate the soluble D-D dimer. On the other hand, the result of plasmin cleavage of FBG or soluble fibrin is another type of measurable marker: fibrin-degradation products (FDPs, fragments X, Y, D and E). Consequently, FDP measurement can also be elevated in the absence of clot, and plasmin is simply cleaving FBG, as in fibrinolysis. Since D-dimer elevation is a possible finding in autoimmune disorders and malignancies at diagnosis [22,23,24], patients with elevated D-dimer should be screened for FDPs to confirm the presence of DIC. The lack of information about FDP level is the main limit of our study [25] and further studies are desirable to explore this specific aspect.

Based on the results of the GIMEMA ALL 0288 protocol [26], steroids have become a standard pretreatment during the induction therapy of ALL. We observed that in most cases, HF occurred in the steroid prephase. The influence of steroids on coagulation is well-known, and HF is described as one of the most frequent findings during steroid therapy [27]. Furthermore, HF during steroid delivery has been reported in other hematological diseases such as chronic lymphocytic leukemia, aplastic anemia and Langerhans cell histiocytosis [28,29,30].

Although the pathogenesis of HF remains unclear, it is reported that tissue factor-like procoagulant activities are detected in ALL cells [31]. A study from Alessio et al. reported that 64% of ALL cells contain cancer procoagulant and showed tissue factor-like activities [32]. Consequently, we would expect greater rates of HF in patients with a larger burden of disease. In contrast, we found that parameters related to disease burden such as baseline WBCc, extramedullary disease and BM blast count did not influence HF. Even the median duration of the steroid prephase did not affect the onset of HF.

In APL, HF is caused by an excess of Annexin II generation, which enhances the t-PA–dependent formation of plasmin on the endothelial-cell surface [33,34]. This protein is also expressed on ALL blasts, leading to hemorrhagic complications [35,36]. Since *BCR-ABL1* is able to indirectly activate Annexin II through Src kinase interaction [37,38], we assume that the steroid-related lysis of *BCR-ABL1*-positive blasts promotes Annexin II activation, which in turn prompts fibrinolysis to take place. These are initial hypotheses which for now lack experimentation to be fully confirmed. There is ultimately a need for a prospective trial to investigate the relationship between BCR-ABL1 protein and Annexin II but also to compare FDPs and D-dimer data, aiming for an in-depth understanding of the mechanism of HF. Moreover, it is essential to design a trial in which steroid prephase days, steroid doses and treatment schedules in both BCR-ABL1-positive and negative groups are uniformed, in order to reduce any possible bias.

Regarding the remission rate, no significant correlation was found; therefore, we can reasonably assume that experiencing HF does not affect the achievement of remission.

The frequency of complication related to HF in ALL is unknown; in our series, we did not observe any clinical sequelae due to the condition of HF, probably as a consequence of a timely instituted prophylaxis.

In this view, a correct management of HF in patients with ALL is not established yet. Antithrombotic and antihemorrhagic prophylaxis for patients using ASP has already been explored in previous studies [39], and there is not unanimity about this issue since the use of FBG concentrates appeared to be associated with a higher risk of venous thromboembolism [40]. In our study, we evaluated patients who have not yet been exposed to ASP and in whom we extensively used both FBG concentrates and FFP, without evidence of thrombotic events.

Some studies showed that HF in adult B-ALL has a low incidence of complications either at diagnosis or during induction treatment [15,41]. Our study confirms that assumption; in fact, the incidence of bleeding was very low, occurring in less than 1% of *BCR-ABL1*-positive and negative patients. On the other hand, FBG concentrates and FFP were frequently administrated in case of severe HF, so this could have possibly reduced the risk of bleeding. However, we can reasonably assume that a correction with FBG concentrates or FFP should be considered to prevent severe bleeding, especially when an invasive procedure is scheduled.

As a matter of fact, our work lacks laboratory methods because of the retrospective nature of the study. Even though we could not identify the mechanism underlying HF, it is significant that almost every BCR-ABL1-positive patient showed it at some point during induction treatment, especially during the steroid prephase.

We believe that our study may have a clinical impact since *BCR-ABL1*-positive patients after diagnosis are usually treated and followed in an ambulatory environment and are often subjected to lumbar punctures. Therefore, monitoring coagulation tests after the start of steroids could be useful to intercept early HF and identify patients at risk of bleeding, in order to carefully plan an invasive procedure and establish prophylaxis on time. However, the correct management of HF in this context should be defined by prospective studies in order to reduce the risk of complications and at the same time avoid unnecessary transfusions.

## 5. Conclusions

In our large retrospective study, we confirmed the tendency of FBG to decrease in B-ALL during the steroid prephase. Even though the ability of steroids to reduce FBG has been described, it is not clear by which mechanism this occurs and we could not exclude the possibility of a primary fibrinolysis. The decrease in FBG did not correlate with the indices of disease activity; conversely, a statistically significant correlation was found with *BCR-ABL1* rearrangement. Therefore, since those patients are often followed in an outpatient environment, we suggest performing a frequent monitoring of coagulation parameters during induction treatment, especially if an invasive procedure is planned to promptly correct the defect and prevent complications.

## Figures and Tables

**Table 1:** Clinical and laboratory characteristics at diagnosis.

*n*	115
Male/Female (%)	60/55 (52/48)
Age, median (range)	56 (18–89)
Age ≤ 65/>65 years	81/34
Congenital FBG disorders or acquired liver disease	0
WBCc × 10^9^/L, median (range)	11.8 (0.840–407)
BM blast cell infiltration %, median (range)	90 (20–100)
Extramedullary disease (%)	38 (33)
*BCR-ABL1* negative/positive (%)	60/55 (52/48)
p190 (%)	38 (70)
p210 (%)	10 (18)
p190/p210 (%)	3 (5)
Protein isoform unknown (%)	4 (7)

FBG: fibrinogen; WBCc: white blood cell count; BM: bone marrow.

**Table 2:** Results in *BCR-ABL1*-positive and negative patients.

	*BCR-ABL1*-Positive	*BCR-ABL1*-Negative
HF steroid prephase (%)	44 (83%)	30 (50%)
G 1–2 (%)	21 (48%)	13 (43%)
G 3–4 (%)	23 (52%)	17 (57%)
Median of days after start of steroid prephase (range)	7 (4–28 days)	7 (3–15 days)
HF after steroid prephase (%)	9 (16%)	4 (7%)
G 1–2 (%)	0	1 (25%)
G 3–4 (%)	9 (100%)	3 (75%)

HF: hypofibrinogenemia; G: grade.

**Table 3:** Univariate analysis.

	HF	Not HF	*p*
Age, median (range)	58 (20–80)	47 (18–89)	0.27
Age ≤65/>65 years	49/25	32/9	0.26
Gender, M/F	43/31	17/24	0.12
WBCc× 10^9^/L, median (range)	12.12 (1–341.5)	10.86 (0.840–407)	0.36
Extramedullary disease (%)	27 (23)	11 (9)	0.44
BM blast-cell infiltration (%), median (range)	90 (20–100)	90 (33–100)	0.31
Days of steroid prephase, median (range)	7 (3–28)	7 (3–10)	0.25
*BCR-ABL1* positivity (%)	44 (38)	11 (9)	0.00158

HF: hypofibrinogenemia; M: male; F: female; WBCc: white blood cell count; BM: bone marrow.

**Table 4:** Description of ISTH-DIC score.

	46 *BCR-ABL1*-Positive	29 *BCR-ABL1*-Negative
No DIC score modification (%)	35 (76%)	28 (96%)
DIC score ≥ 5 at diagnosis (%)	4 (8, 7%)	0
DIC score ≥ 5 at start of CHT/TKI (%)	7 (15, 3%)	1 (4%)

DIC: disseminated intravascular coagulation; CHT: chemotherapy; TKI: tyrosine kinase inhibitors.

## Data Availability

The data presented in this study are available on request from the corresponding author.

## Supplementary Materials

The following supporting information can be downloaded at: https://www.mdpi.com/article/10.3390/jcm11071776/s1, Figure S1: Liver function tests; Figure S2: INR levels in patients with HF; Figure S3: Difference between *BCR-ABL1* positive and negative patients regarding HF during steroid pre-phase; Figure S4: Difference between *BCR-ABL1* positive and negative patients regarding HF during induction treatment; Figure S5: ISTH-DIC score comparison between the start of steroid pre-phase and the start of CHT/TKI in *BCR-ABL1* positive patients.
