# Supplementary material for: In BCR-ABL1 Positive B-Cell Acute Lymphoblastic Leukemia, Steroid Therapy Induces Hypofibrinogenemia
Source: J Clin Med. 2022 Mar 23;11(7):1776. doi: 10.3390/jcm11071776 (PMC8999266; doi:10.3390/jcm11071776)
Supplement: Supplementary file 1 [file jcm-11-01776-s001.zip › Figure S1.pdf]

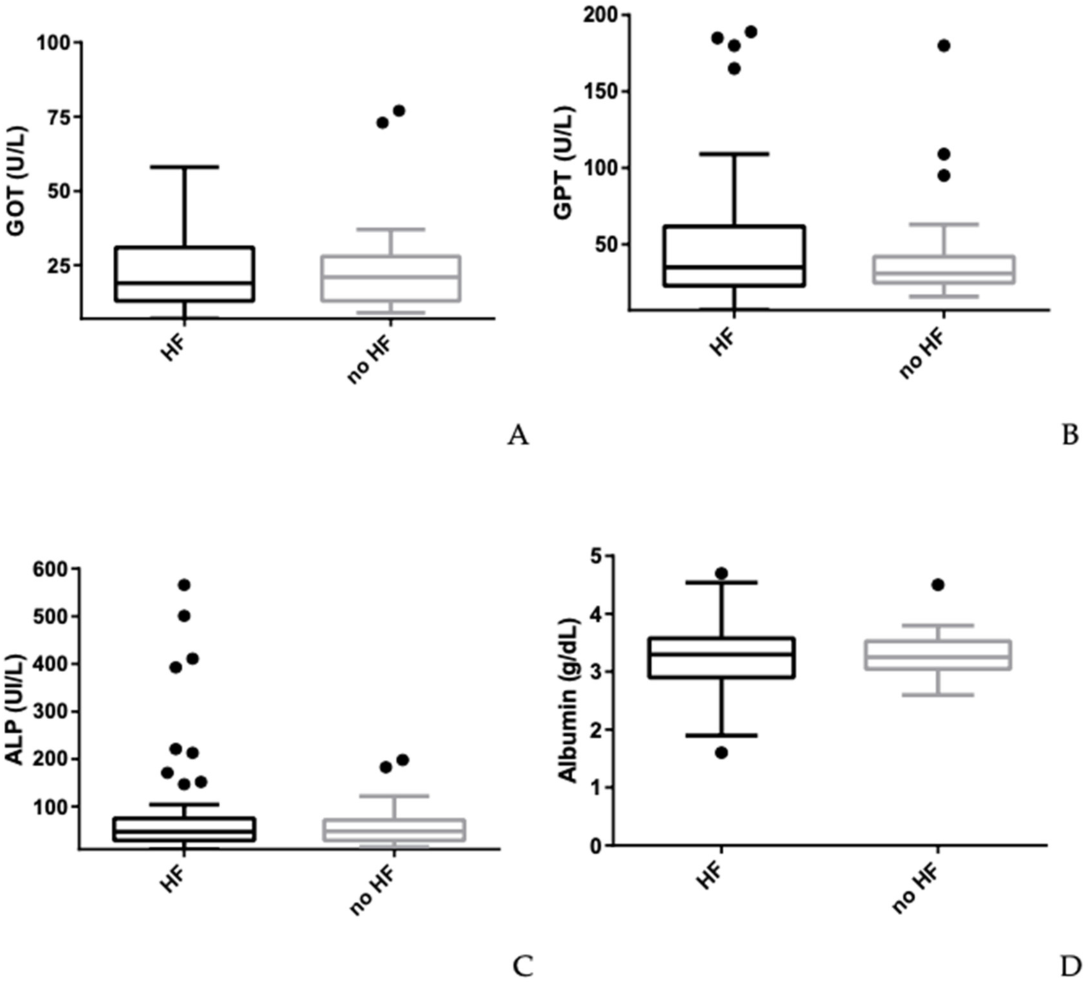

Figure S1. Liver function tests. (**A**) Correlation between patients who experienced HF and patients who did not regarding GOT levels in univariate analysis $p = 0.87$. GOT: serum glutamic oxaloacetic transaminase; HF: hypofibrinogenemia. (**B**) Correlation between patients who experienced HF and patients who did not regarding GPT levels in univariate analysis $p = 0.66$. GPT: serum glutamic pyruvic transaminase; HF: hypofibrinogenemia. (**C**) Correlation between patients who experienced HF and patients who did not regarding ALP levels in univariate analysis $p = 0.97$. ALP: alkaline phosphatase; HF: hypofibrinogenemia. (**D**) Correlation between patients who experienced HF and patients who did not regarding Albumin levels in univariate analysis $p = 0.74$. HF: hypofibrinogenemia. The dots outside the whiskers represent the outliers.
