# Supplementary material for: In BCR-ABL1 Positive B-Cell Acute Lymphoblastic Leukemia, Steroid Therapy Induces Hypofibrinogenemia
Source: J Clin Med. 2022 Mar 23;11(7):1776. doi: 10.3390/jcm11071776 (PMC8999266; doi:10.3390/jcm11071776)
Supplement: Supplementary file 1 [file jcm-11-01776-s001.zip › Figure S2.pdf]

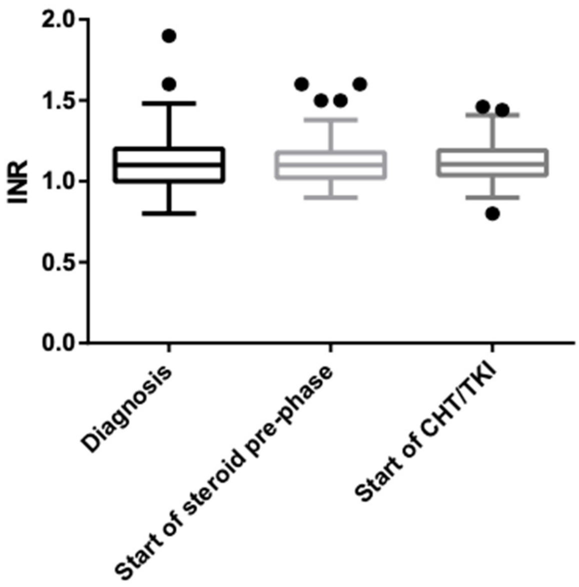

Figure S2. INR levels in patients with HF. In univariate analysis no significant difference was detected among the three timepoints ($p = 0.89$). INR: international normalized ratio; HF: hypofibrinogenemia; CHT: chemotherapy; TKI: tyrosine kinase inhibitors. The dots outside the whiskers represent the outliers.
