# Supplementary material for: In BCR-ABL1 Positive B-Cell Acute Lymphoblastic Leukemia, Steroid Therapy Induces Hypofibrinogenemia
Source: J Clin Med. 2022 Mar 23;11(7):1776. doi: 10.3390/jcm11071776 (PMC8999266; doi:10.3390/jcm11071776)
Supplement: Supplementary file 1 [file jcm-11-01776-s001.zip › Figure S3.pdf]

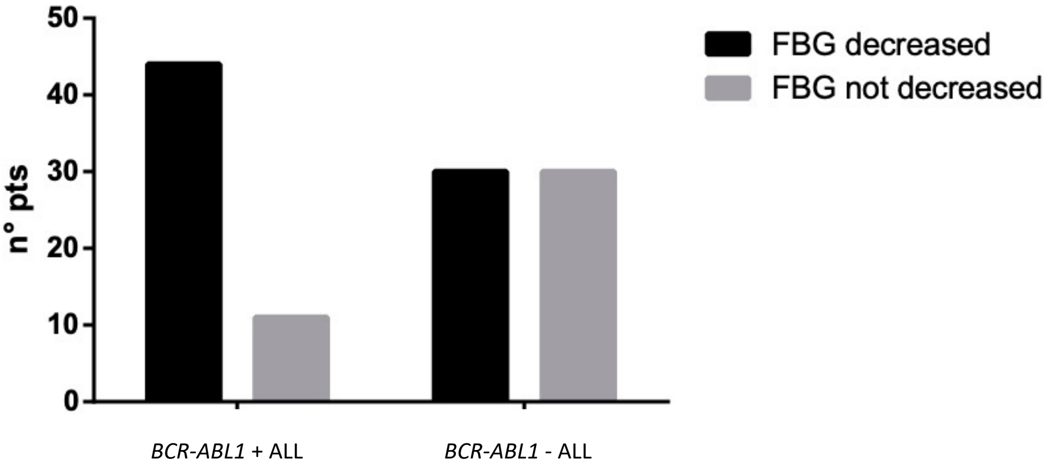

Figure S3. Difference between *BCR-ABL1* positive and negative patients regarding HF during steroid pre-phase. Pts: patients; ALL: acute lymphoblastic leukemia; FBG: fibrinogen. *BCR-ABL1* positive: FBG decreased in 44 patients, FBG not decreased in 10 patients. *BCR-ABL1* negative: FBG decreased and not decreased in 30 patients. Correlation between HF and *BCR-ABL1* positivity during steroid pre-phase: $p = 0.00158$.
