# Supplementary material for: In BCR-ABL1 Positive B-Cell Acute Lymphoblastic Leukemia, Steroid Therapy Induces Hypofibrinogenemia
Source: J Clin Med. 2022 Mar 23;11(7):1776. doi: 10.3390/jcm11071776 (PMC8999266; doi:10.3390/jcm11071776)
Supplement: Supplementary file 1 [file jcm-11-01776-s001.zip › Figure S4.pdf]

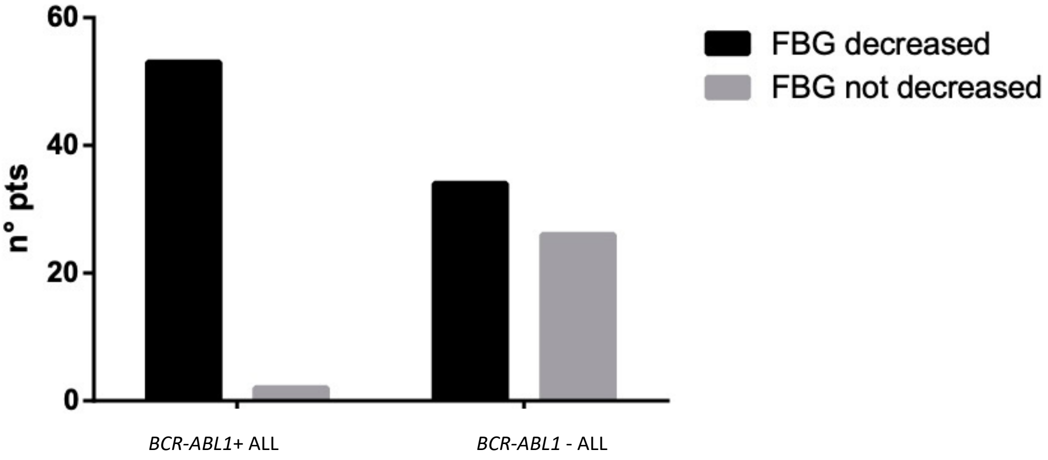

Figure S4. Difference between *BCR-ABL1* positive and negative patients regarding HF during induction treatment. Pts: patients; ALL: acute lymphoblastic leukemia; FBG: fibrinogen; HF: hypofibrinogenemia. *BCR-ABL1* positive: FBG decreased in 53 patients, FBG not decreased in 2 patients. *BCR-ABL1* negative: FBG decreased in 34 patients and not decreased in 26 patients. Correlation between HF and *BCR-ABL1* positivity during induction treatment: p <0.0001.
