# Supplementary material for: In BCR-ABL1 Positive B-Cell Acute Lymphoblastic Leukemia, Steroid Therapy Induces Hypofibrinogenemia
Source: J Clin Med. 2022 Mar 23;11(7):1776. doi: 10.3390/jcm11071776 (PMC8999266; doi:10.3390/jcm11071776)
Supplement: Supplementary file 1 [file jcm-11-01776-s001.zip › Figure S5.pdf]

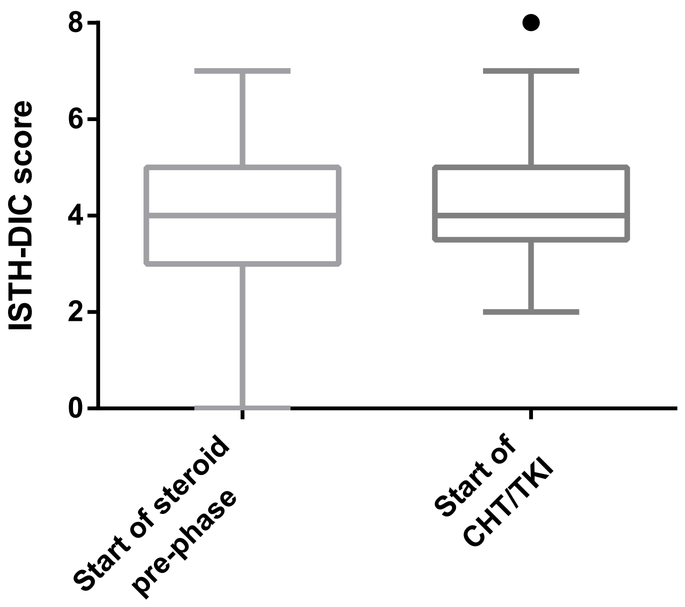

Figure S5. ISTH-DIC score comparison between the start of steroid pre-phase and the start of CHT/TKI in *BCR-ABL1* positive patients (p = 0.26). ISTH-DIC: International Society of Hemostasis and Thrombosis- Disseminated Intravascular Coagulation; CHT: chemotherapy; TKI: tyrosine kinase inhibitors. The dot outside the whiskers represents the outlier.
